# Rehabilitation Technology: Assistance from Hospital to Home

**DOI:** 10.1155/2019/1431509

**Published:** 2019-06-02

**Authors:** Ester Martinez-Martin, Miguel Cazorla

**Affiliations:** RoViT, University of Alicante, P.O. Box 99, 03080 Alicante, Spain

## Abstract

Rehabilitation is essential for disabled people to achieve the highest level of functional independence, reducing or preventing impairments. Nonetheless, this process can be long and expensive. This fact together with the ageing phenomenon has become a critical issue for both clinicians and patients. In this sense, technological solutions may be beneficial since they reduce the costs and increase the number of patients per caregiver, which makes them more accessible. In addition, they provide access to rehabilitation services for those facing physical, financial, and/or attitudinal barriers. This paper presents the state of the art of the assistive rehabilitation technologies for different recovery methods starting from in-person sessions to complementary at-home activities.

## 1. Introduction

According to the World Health Organization (WHO), about 15% of the world's population suffers some form of disability. Due to the ageing phenomenon and the prevalence of chronic diseases such as epilepsy, cancer, or mental health disorders, this percentage has incessantly increased. This fact leads to a growing demand for rehabilitation services since they play an important role in enhancing functioning, reinforcing the person's autonomy, and improving the patient's quality of life [1, 2]. This demand far exceeds availability in terms of rehabilitation professionals (i.e., occupational therapists, physiotherapists, and speech therapists) such that the density of those professionals is greatly below the threshold required for providing adequate services (approximately a tenth of that required) [3, 4]. Additionally, different barriers like low-income deny the access to the rehabilitation services required to live in health, comfort, and dignity. These deficiencies could be overcome with technology, reducing the need for formal support services, the time and physical burden for caregivers, and, consequently, their cost [5, 6].

In this context, the key to technology success depends on its functionality and adaptability to the user's needs and environment. However, rehabilitation is a broad concept covering a wide range of responses to disability. Generally speaking, rehabilitation can be defined as the step-by-step process designed to reduce disability and to optimise functioning in individuals with health conditions, enabling them to better interact with their environment. For that, rehabilitation commonly includes three aspects:

- Physical, to regain strength, mobility, and fitness
- Occupational, to relearn the person's daily activities
- Speech-language, to recover communication skills (i.e., speaking, understanding, reading, or writing)

The duration of the rehabilitation can vary depending on several factors such as the patient's impairment level, the therapy intensity, or the individual activity and participation. For that reason, new ways without compromising patient wellbeing have been proposed. So, three different modalities can be found in the literature: (1) the in-person rehabilitation, where patients performs their program in presence of a therapist in an inpatient facility; (2) the combined in-person and at-home rehabilitation, where in-person rehabilitation takes place in an outpatient facility and is aided with at-home programs such that patients perform some therapeutic exercises prescribed by the clinician at home; and (3) the at-home rehabilitation, suitable for those requiring minor assistance or support, where a tailored therapy takes place entirely at home.

Focusing on the individual's functioning, the technological solutions developed up to date have mainly aimed to physical recovery since mobility plays a main role in the independence and confidence of disabled people. More recently, research in occupational rehabilitation has emerged in response to Alzheimer's disease and neurocognitive impairments.

This paper addresses the state-of-the-art assistive technologies for rehabilitation from the hospital to in-home programs. Despite its great importance in disabled people recovery, devices designed to replace the impaired limb (e.g., prosthetics and artificial limbs [7, 8, 9, 10] or smart wheelchairs [11, 12, 13]) are not covered in this work.

## 2. In-Person Rehabilitation

One application of technology can be found as a support tool in the rehabilitation process. They help clinicians evaluate quantitatively the patient's performance and progress while providing consistent training, specially for extended periods of time. This results in an increase in therapy access and a health-care cost reduction.

In this sense, Robotics has met this demand with a wide range of assistive products. For example, Andago [14] is a tool for overground gait training, bridging the gap between treadmill-based and free walking. With this technology, the patient's fear of falling is considerably reduced while therapists focus on the therapy since they do not have to secure the patient. In a similar way, the G-EO System [15] assists therapists in patient's motor recovery and, more specifically, in teaching patients walking again. Unlike the previous system, G-EO moves the patient's legs when necessary to help the patient's brain form new neuroplasticity pathways to replace the ones damaged by injury or disease. Kim and Deshpande presented in [16] HARMONY an upper-body robotic exoskeleton for rehabilitation. This exoskeleton provides natural coordinated motions on the shoulder for patients suffering from spinal and neurological injuries, including a wide range of motion and controllability of force and impedance. Several devices have been also developed for hand rehabilitation (e.g., [17, 18, 19, 20]).

Although these robotic devices aid therapists in providing effective repetitive training and quantitative evaluation of patient's progress, it is necessary to integrate any mechanism that makes rehabilitative exercises fun, challenging, and engaging. In this context, virtual reality (VR) and video game can fill the gap. That is, computer-based programs designed to simulate real-life objects and events in an attractive environment may engage patients to stage on track. In fact, the use of this kind of systems has been shown to be an effective mean for rehabilitation treatments since they offer clinicians the ability to control and grade tasks to challenge the user while providing them with an enriched environment to achieve high user's engagement [21, 22].

**Figure 1:**
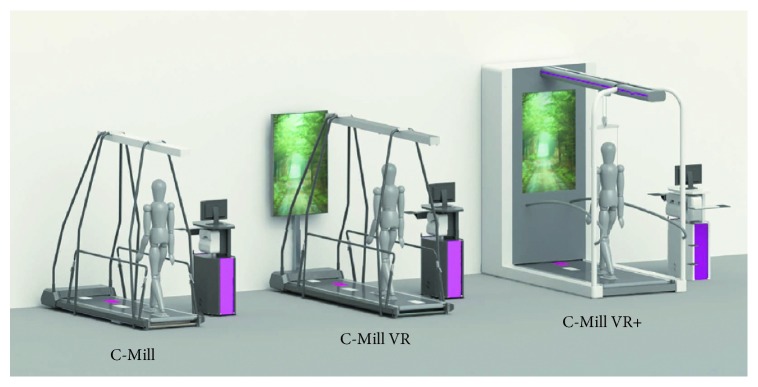
C-Mill [23] models: C-Mill, C-Mill VR, and C-Mill VR+.

From this starting point, a treadmill can be combined with VR technology. This is the case of C-Mill [23], a treadmill developed to train and assess patient's gait and balance for a safe daily walk. It comes in three models: C-Mill, C-Mill VR, and C-Mill VR+ (Figure 1). Although he VR and VR + models use VR elements to stimulate and challenge patients, their final goal is different. That is, the C-Mill VR is aimed at training automated movements and dual tasking, whilst the C-Mill VR+ is a comprehensive solution for early to late rehabilitation with balance and body weight support.

Another example is Lokomat [24], an exoskeletal robot with VR intended to highly intensive physiological gait rehabilitation for severely impaired neurological patients. As previously discussed, several parameters (e.g., speed, loading, or robotic support) can be adjusted to optimally shape the intensity of the therapy. Another device exploiting this combination is Riablo [25]. Unlike the previous systems, Riablo uses games with a treadmill. So, this orthopedic rehabilitation platform allows patients to play video games while performing the exercises recommended by their physiotherapist. In this case, high-precision sensors must be worn by patients to properly measure their body movements. In addition, a web application helps clinicians plan the rehabilitation exercises as well as the therapeutic video games to keep the patient motivated and continuously improving.

**Figure 2:**
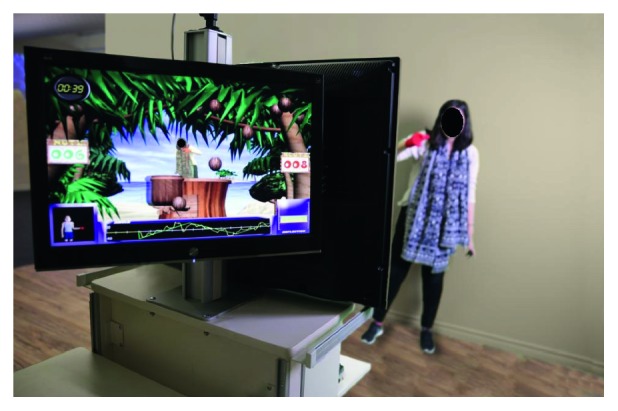
IREX [26] sample of therapeutic session with a monitoring screen for clinicians and another one for the patient.

Going a step further, GestureTek developed IREX (Immersive Rehabilitation EXercise) [26], a virtual reality therapy system that uses a camera connected to a computer with immersive video gesture control technology to place patients into virtual environments as depicted in Figure 2. With more than 20 applications to choose, clinicians can set up a therapy program for the current patient's level of ability and recovery. This variety together with real-time auditory and visual feedback keeps the patient's motivation, resulting in an improved participation and focus on the rehabilitation exercises. Moreover, the data gathered from the performed physical tasks (e.g., balance, rotation, exertion, timing, or abduction) provide clinicians with information about the patient's range of motion, frequency of successful exercises, and number of repetitions completed, quantitatively measuring the patient's progress. IREX's efficacy has been proven for different impairments [27, 28, 29, 30].

Similarly, SeeMe [31] was developed. This PC-based platform was also designed to aid the rehabilitation process and track the patient's progress. For that, a series of therapeutic tasks with different levels and parameters (e.g., frequency and intensity) provide a motivating environment to evaluate the patient's range of motion, quality of movement, postural control, strength, proprioception, perception, endurance, memory, and divided attention. In this way, the therapist can plan a tailored set of tasks adapting them to the patient's needs in real time. In addition, a preview of all the collected statistics are available to the therapist such that they can make objective evaluations of the rehabilitation progress and proper clinical decisions.

## 3. Combined In-Person and At-Home Rehabilitation

A proper recovery is composed of two essential components: an in-clinic supervised learning and a recommended home exercise routine. In this way, the home exercise program may increase therapeutic gains. Note that this exercise routine must be designed to be practical, accessible, feasible, and easy to complete to maximise the patient's efforts without instruction. With that purpose, some technological solutions are designed to provide patients with assistance in both in-person and at-home rehabilitation. So, an in-clinic supervised learning facilitates the at-home therapy, which continuously reports the patient's progress to their care specialist.

One of the early approximations was the incorporation of Nintendo Wii in the rehabilitation process [32, 33]. Despite its success, this device was not aimed at rehabilitation. Nevertheless, it opened a new research line. This is the case of the jintronix rehabilitation system (JRS), a virtual rehabilitation platform for physical therapy. From its first attempt as an adaptive game consisting of a Nintendo Wii remote and a web application, JRS has evolved to an engaging VR tool to exercise while monitoring and tracking the patient's progress on a session-to-session basis through their physical therapy.

**Figure 3:**
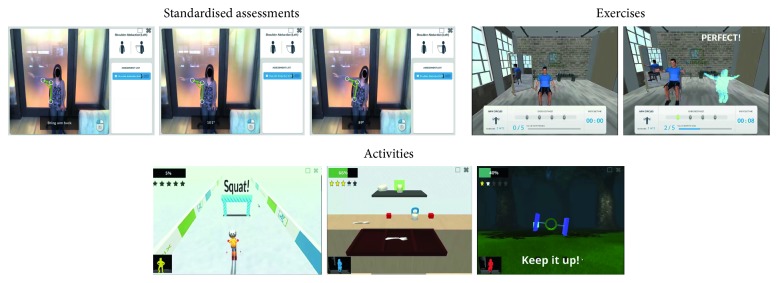
Jintronix's [34] functionalities.

With that aim, three different functionalities have been implemented (Figure 3):

- Standardised assessments: this functionality offers an accurate measurement of user's movements based on its motion library with parameterised measurements and its implementation of body compensation to reduce assessment errors
- Exercise platform: this functionality shows a virtual gym where a monitor is performing the exercise indicated at the bottom of the screen together with a digital user's representation who is continuously getting visual feedback about their performance
- Activities: this functionality includes a wide range of virtual reality environments representing real-life scenarios to challenge the user to work on their motor, balance and mental skills.

These functionalities become JRS into a complementary tool to in-person therapy as well as a virtual therapist for at-home rehabilitation. In both cases, a therapist subscribes a set of exercises and activities (graded according to the patient's level) to stimulate their recovery. The remote patient's monitoring is possible thanks to the JRS register that provides care specialists with a detailed patient's progress in terms of session durations, movement accuracy, and progress with respect to previous performances and reaction time. In this way, the therapist can view and evaluate the patient's progress and adapt their activity program to their needs.

Thinking of movement learning at neural level led to IKKOS [35], a VR solution for able-bodied people and those with mobility deficits. Its core idea is learning by imitation. So, an audiovisual combination is used for training the patient's nervous system to move correctly. It is noteworthy that, although IKKOS can be used in the clinic and at home, the movement learning is enhanced in healthcare facilities where an immersive experience takes place.

**Figure 4:**
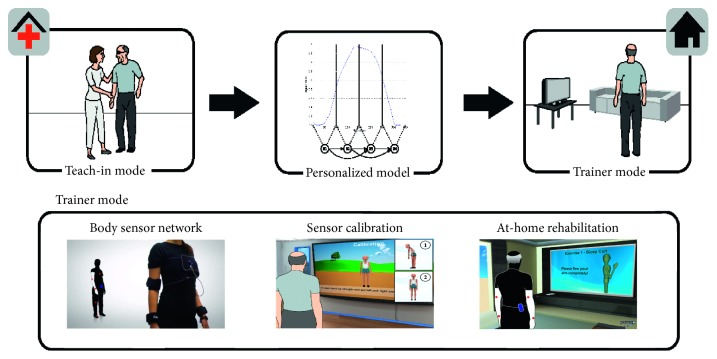
PAMAP [36] workflow for personal physiotherapist functionality. Firstly, the user guided by a specialist performs a series of exercises to record a reference motion profile in the teach-in mode. After that, in the home trainer mode, the user must locate the body sensor network in the proper positions and then calibrate it. Finally, the user performs the proposed exercise while being monitored and warned about their movements. These data are remotely available for the specialist to make the corresponding recommendations for the continued at-home rehabilitation process.

With the purpose of diagnosing disease at an early stage and encouraging people to a healthy activity level, the PAMAP (physical activity monitoring for aging people) assistive system was developed [36]. So, a body sensor network located at selected positions on the upper and lower body is in charge of capturing the patient's motion and vital signs. Note that a sensor calibration session is required for gathering accurate data as depicted in Figure 4. Among PAMAP's functionalities, a personal physiotherapist has been implemented. In the teach-in mode, the therapist works with the user to record a user's reference motion. In the home trainer mode, the PAMAP system monitors and promotes repetitive limb exercises typically prescribed to stroke patients to regain or retain mobility. The system downs repetitions and evaluates correctness of the movement, frequency, range, and amplitude based on the user's reference motion. When an incorrect movement is performed, the system warns the user providing graphical help to correct it. All the measurements and session data are collected, processed, and stored in the user's electronic health record (EHR) that is remotely available through PAMAP web application. In this way, a rehabilitation specialist can get a solid ground for making recommendations for the continued rehabilitation process.

The need of combined therapies led Gomez-Donoso et al. [37] to develop a robotic multisensor platform for motor and cognitive rehabilitation. So, with the use of 3D sensors, colour cameras, microphones, eye tracker, and a tactile interface, an interactive interaction provides the patient with a tool for performing rehabilitation tasks such as memory and brain games, physical activities, or other therapy programs. Thus, the first rehabilitation sessions are performed with the help of a therapist and, after that, at-home rehabilitation takes place.

## 4. At-Home Rehabilitation

Going a step further, autonomous rehabilitation systems could encourage and monitor the rehabilitation at home always under the remote therapist's supervision. Thus, these kinds of systems are appropriate for keeping improvement when specialist visits end, complementing in-person sessions, and/or avoiding problematic patient's displacement to healthcare facilities.

**Figure 5:**
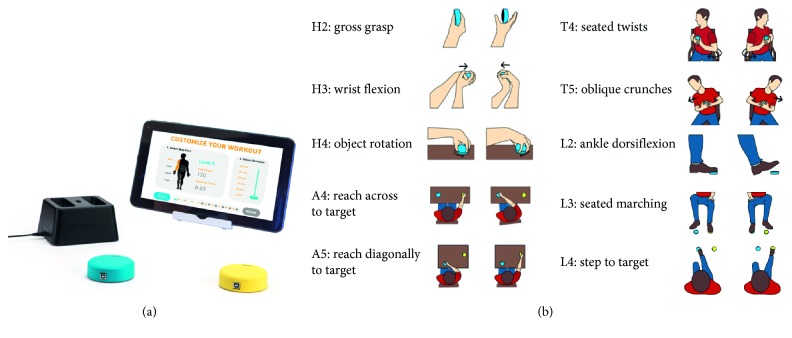
FitMi [38] system on the left and some of the 40 full-body exercises.

Under the assumption that repetition is the key to a successful stroke recovery, the home-therapy tool FitMi was proposed [38]. This solution consists of two wireless pucks and a therapy app with 40 full-body exercises (Figure 5). Each exercise can be performed at 10 difficulty levels. This together with the selection of body part to work on (i.e., hand, arm, core, or legs) allows FitMi to gradually tailor the exercise program to be performed according to the patient's stage of recovery. For that, complete repetitions are measured and compared to the previous performances. These measurements are also used to provide the patient with immediate visual, auditory, and haptice feedback as well as daily summaries and long-term performance trends.

**Figure 6:**
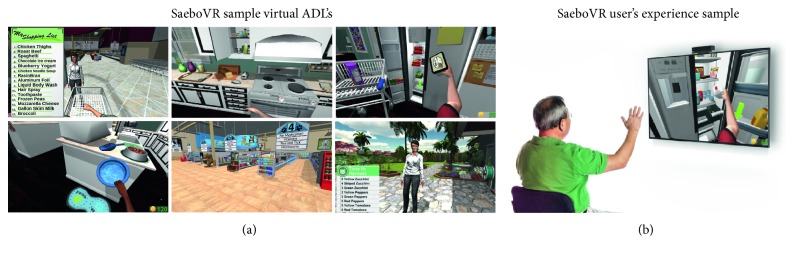
(a) SaeboVR's virtual worlds for the challenges: grocery shopping, preparing breakfast/dinner, putting away groceries, pet feeding, pet shopping, and garden harvesting, (b) together with a user's experience sample [39].

**Figure 7:**
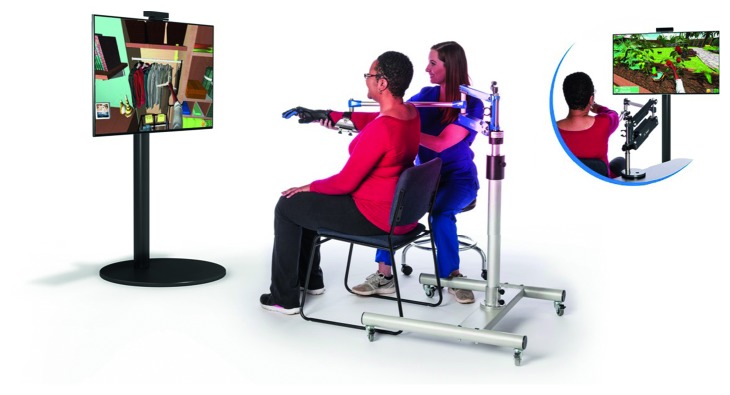
SaeboVR combined with SaeboMAS, SaeboReJoyce, and SaeboGlove for shoulder and hand recovery [39].

On their behalf, Saebo Inc. proposed SaeboVR [39], a nonimmersive VR rehabilitation system involving daily functional activities. As previously discussed, a virtual world simulating real-life challenges is projected on the screen while a Microsoft Kinect sensor is monitoring the patient's impaired arm movements for picking up, manipulating and/or transfering various virtual objects (Figure 6). For that, four islands have been designed based on the kind of daily activities to be achieved: fun with food, puppy playtime, garden bounty, and home stretch. Each island includes two to three activities in sequence that should be repeated a total of four times before moving to the next island. Additionally, a balls and boxes exercise is performed at the beginning and ending of each island for the user's progress monitoring. All these tasks can be tailored to test and train user's cognitive and motor skills such as endurance, speed, range of motion, coordination, timing, and cognitive demand (e.g., visual-spatial planning, attention, or memory). This progress is graphically displayed and also registered in a clinical provider dashboard for further analysis. Note that additional technologies such as SaeboMas, SaeboReJoyce, or SaeboGlove can be integrated in this virtual environment for the shoulder and/or hand treatment (Figure 7).

**Figure 8:**
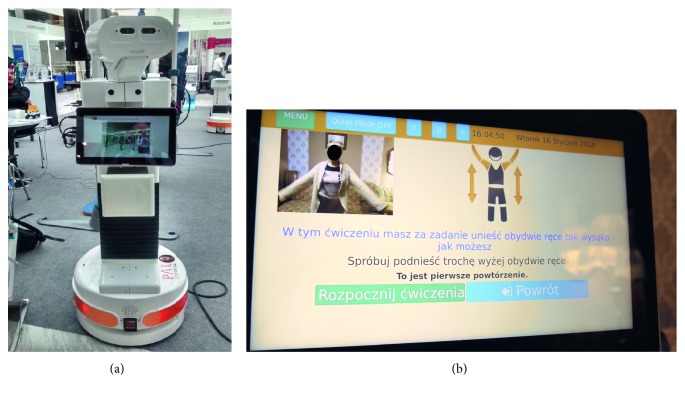
ENRICHME interactive mobile robot (a) and its service of physical exercise monitoring (b).

Exercise and staying active are an important part of rehabilitation for chronic diseases [40, 41]. In this regard, the EU-funded project ENRICHME (ENabling Robot and assisted living environment for Independent Care and Health Monitoring of the Elderly) [42] developed an interactive mobile robot in an assisted living environment for the provision of advanced user services, integrated within a domestic RFID ecosystem. Addressing the progressive decline of cognitive and motor capacity in the ageing population, among its typical services, includes exercise remainder and monitoring, as depicted in Figure 8. Thus, after the reminder and user approval for physical activity through the robot display, the total exercise time is chosen from the available options. Then, the robot describes acoustically and graphically exercise by exercise while showing its camera input. However, no feedback information about the user's performance is provided.

Costa et al. proposed PHAROS [43], an interactive robot system aimed at staying active as part of rehabilitation for chronic diseases. With that aim, a recommender system stores the specialist home exercise and reminds the patient their daily sessions. After a visual and auditory exercise description, the robot platform monitors the user's performance, registering the user's movements and exercise completeness. PHAROS uses this information to automatically adapt the home exercise program focusing on the impaired limbs requiring more recovery.

## 5. Discussion and Conclusions

Almost a sixth of the world's population suffers some type of disability. This fact has led to an unceasing demand for rehabilitation services, impossible to satisfy with the limited healthcare professionals. In this context, technology can be a solution.

Starting from the labor-intensive rehabilitation processes, the early rehabilitation technology was focused on robotic devices as a tool for repetitive movement training. In addition, these devices allow clinicians to quantitatively measure the patient's performance and progress. Nonetheless, the monotony of rehabilitation sessions resulted in a loss of patient's interest and, consequently, in a poor improvement. As a consequence, new mechanisms like virtual reality or video games were integrated to make the rehabilitation exercises fun, challenging, and engaging.

From the rehabilitation's point of view, it is important to take intensive and continuous therapeutic exercise for a successful patient's recovery. This requirement can be satisfied when therapy is not only taken place in hospitals but also at home. Along this line, research responded with hybrid systems. So, the in-person sessions allow the patients to be familiar with the tool and set configuration parameters when necessary, while the at-home sessions provide the patients with the tailored exercise program set by the specialist. In this last case, the patients usually get immediate feedback on the performed exercises and, at the same time, these data are remotely available to the clinicians at any time. Going a step further, autonomous rehabilitation systems are being developed to prevent impairments, specially in the case of elderly, and to in reach for those with travelling difficulties.

It is noteworthy that despite the adaptation to the patient's needs, most of the existing literature is focused on physical therapy. However, the ageing phenomenon and some diseases like Alzheimer are demanding new assistive solutions for occupational and speech-language therapies. Although some efforts have been done in this sense, there is still a long way to go.
